# Factors Correlated with Home Gardening in Gauteng Province, South Africa

**DOI:** 10.3390/ijerph18052737

**Published:** 2021-03-08

**Authors:** James Wabwire Oguttu, Tulisiwe P. Mbombo-Dweba, Jabulani R. Ncayiyana

**Affiliations:** 1 Department of Agriculture and Animal Health, College of Agriculture and Environmental Sciences, University of South Africa, Johannesburg 1709, South Africa; Mbombtp@unisa.ac.za; 2 Department of Public Health Medicine, School of Nursing and Public Health, College of Health Sciences, University of KwaZulu-Natal, Durban 4041, South Africa; NcayiyanaJ@ukzn.ac.za

**Keywords:** home food gardens, growing food or vegetables, household food security, livelihood strategy, food security policy

## Abstract

Background: In addition to increasing access to fresh and affordable produce, home gardening enhances food security. This notwithstanding, there is no evidence of studies that have investigated factors correlated with home gardening in Gauteng Province (GP), South Africa. The present study investigated home gardening across the GP. Methods: Retrospective data of residents of GP (*n* = 30002) collected by the Gauteng City Region Observatory were used. A binary logistic regression was employed to determine factors correlated with home gardening. Results: Overall participation in home gardening was low (12.37%). If a respondent was a resident of the poorest areas, resided in a house received under the Rural Development Programme, had a borehole/well as the main source of water, belonged to a social club, received a social grant, was >65 years, and rated his/her health as poor, then they were more likely to participate in home gardening. Factors that were negatively correlated with home gardening included if the respondent rented from private individuals and if the respondent’s health status prevented him/her from doing daily work. Conclusion: The low participation levels in home gardening observed suggest the failure of the current policies geared at fostering home gardening in the province. Policy makers and relevant authorities should target identified groups to improve participation in home gardening.

## 1. Introduction

Food-insecure households have been defined as families that are unable to access and afford nutritious food that meets their dietary needs for a productive and healthy life. Food insecurity is a global problem that has been getting worse with an upward trajectory since 2014. In 2019, the Food and Agriculture Organization (FAO), along with other international organisations [1], estimated that approximately 750 million people experienced severe levels of food insecurity, 130 million experienced acute hunger, and 2 billion people did not have regular access to safe and nutritious food. The upward trend of food insecurity has the potential to jeopardise efforts to achieve zero hunger by 2030 [2]. Moreover, with the advent of coronavirus disease 2019 (COVID-19), the food insecurity situation is expected to worsen [1,2]. In fact, it is estimated that due to the COVID-19 pandemic, an additional 83 to 132 million people are likely to be at risk of food insecurity [1]. It is noteworthy that the majority of people affected by food insecurity live in Africa, Asia, and Latin America [1].

Although South Africa is generally considered to be a food-secure country, this is only at the national level. Disparities in food insecurity in local communities and households do exist. It is estimated that up to 20% of local households in South Africa experience food insecurity [3]. Various factors have been associated with food insecurity, and they include poverty, extensive unemployment, insufficient food production, degradation of natural resources, and increased food prices. Without mitigation strategies, these factors can push vulnerable households further into hunger [3].

To enhance food production and alleviate food insecurity at the household level, the South African government has implemented several land, food, and agricultural policies. It has also implemented several programmes purposed to enhance food production and income-generating opportunities. The Agriculture and Land Reform Policy (ALRP), South Africa Integrated Food Security Strategy (SAIFSS), and the Comprehensive Agricultural Support Programme (CASP) are among some of the policies and programmes that the South African government has initiated and/or implemented to help improve the food security status of the country, and also uphold the constitutional rights of every South African to have access to sufficient food [3].

Home gardens are one of the multiple strategies available to combat the scourge of food insecurity in communities with limited resources and institutional support. Home gardening is considered to be an effective mitigation strategy to combat household food insecurity [4]. Moreover, home gardening is an important food security intervention within the SAIFSS programme [3]. According to Rammohan, Pritchard, and Dibley [5], home food gardens are cost effective and are one of the most sustainable approaches for fighting food insecurity and malnutrition in under-resourced communities. As a source of food, home gardens are able to alleviate both food insecurity and malnutrition [4,6].

By providing households with easy access to fruit and vegetables, home food gardens improve the dietary quality of households [6,7]. Home food gardens have also been associated with increased availability and access to a variety of vegetables, which leads to increased dietary diversity [5,6]. Dietary diversity has been defined as the consumption of a variety of food groups over a given reference period [8]. Moreover, increased dietary diversity is a strong predictor for positive nutritional outcomes [6]. 

Home food gardens have the potential to improve food security in both urban and rural areas [4]. In rural Myanmar and Australia, food gardens improved food security amongst vulnerable households. This observation was also confirmed in a study that was conducted in rural Nkonkobe Municipality in the Eastern Cape, South Africa [9].

Participating in home gardening has also been reported to be beneficial in developed and developing countries. For example, a review of studies conducted in both North America and Sub-Saharan Africa concluded that participating in urban gardening has a positive influence on food security [7].

The contribution of home food gardens is not only limited to food and nutrition security. Available evidence suggests that home gardens also have the potential to generate income and, thus, contribute to the economic welfare of the participating households [4,10]. Furthermore, through reduced expenditure on food, because of the increased reliance on food produced in one’s own garden, home food gardens contribute to the economic welfare of families [7]. Increased income associated with home food gardens has been reported in several studies that were reviewed by Suri [11] and Galhena [4]. Both sets of authors were able to conclude that income can be generated from the sale of home food garden produce, turning home gardening activities into small cottage industries.

Home food gardens have also been reported to contribute to job creation. For example, in a study conducted in Langa Township, Cape Town, South Africa (SA), 38% of the participants were employed in home food gardens.

Despite the benefits of home food gardens, existing literature suggests that participation in home gardens is low in some parts of South Africa [10,12] and continues to decline at a national level [12]. For example, a study that was conducted in the Western Cape Province of South Africa by Philander et al. [10] observed that only 38% of respondents participated in home gardening. Meanwhile, a study conducted at national level by Statistics South Africa [12] reported that only 15.6% of households in South Africa participated in agricultural activities. Constraints to home gardening include land ownership [9,13], low socio-economic status, low educational status, access to advisory services [9], access to water [4], and female headed households [9,14].

However, past studies on home gardens in South Africa have involved small sample sizes, which limits generalisation to the larger population [9,10]. In addition, the data provided by Statistics South Africa lack specific information on the level of home gardening and, instead, report on agricultural activities in general and not specifically home food gardening [12]. 

The Gauteng Department of Agriculture and Rural Development (GDARD) is one of the provincial government departments that has developed “Homestead Food Gardens”, and the purpose of the project is to promote growing of vegetables and, thus, contribute to realising food security. However, there is no evidence of studies that have assessed the extent of involvement in home gardening (growing food or vegetables in their homesteads) at provincial level in the Gauteng Province (GP) of South Africa. Therefore, this study used retrospective data collected by the Gauteng City Region Observatory (GCRO) in 2015 to investigate (1) the level of participation in home gardening and (2) factors correlated with home gardening in the study area.

Findings of this study can be used by policy makers to design intervention programmes aimed at improving participation levels in home gardening among the residents of the study area. This has the potential to improve food security in the study area.

## 2. Conceptual Framework

**Figure 1:**
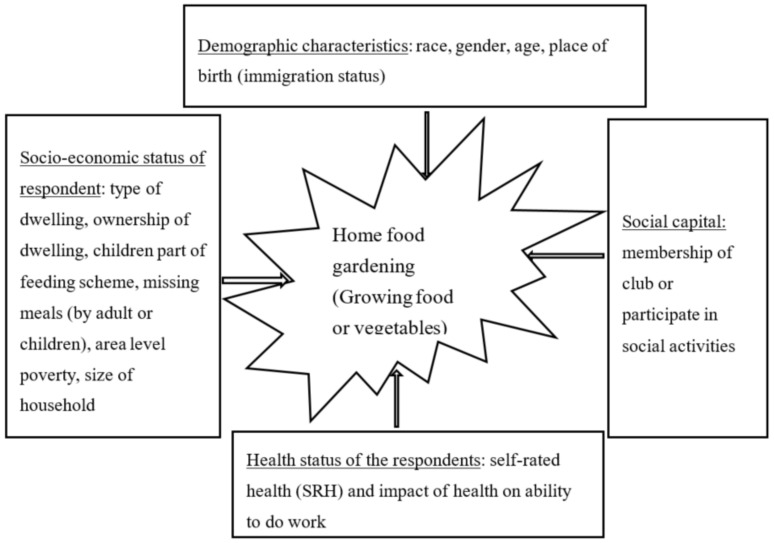
Conceptual framework of the relationship between home gardening (growing food in the backyard of the homestead) (dependent variable) and the covariates.

The theoretical justification of the relationships we investigated are demonstrated in the conceptual framework presented in Figure 1. The study is underpinned by the theory that presupposes that growing food or vegetables (also known as home food gardening) is influenced by the following covariates: social capital, socio-economic factors, demographic factors, and the health status of the respondents.

## 3. Materials and Methods

### 3.1. Study Setting

The study was conducted among residents of Gauteng Province (GP), which is the smallest of the nine provinces in South Africa. The province is located in the central north-eastern part of South Africa and has an area of 18,178 km^2^ [15,16]. According to the 2019 mid-year population estimates, it has an estimated population of 15,176,115, which is roughly 25.82% of the entire population of the country [17]. Only areas that fall under the GCRO were sampled.

### 3.2. Study Design and Study Population

This study adopted a cross-sectional study design, weighted to be representative of the general population of Gauteng Province that was employed by the Gauteng City Region Observatory (GCRO)—the originator of the data. Randomly selected residents of GP that were included in the 2015 wave of the Quality of Life (QoL) survey by the GCRO constituted the study population. Identification of participants (*n* = 3002) involved in the study was done using stratified multistage random sampling using the 2011 wards (*n* = 508) as the strata. In addition, probability proportional to size (PPS) was employed with the power allocation rule to sample enumerator areas (EAs). A minimum of 30 respondents per ward were selected from non-metro wards and 60 were selected from metro wards.

### 3.3. Data Collection and Sources of Data

The GCRO conducts the QoL survey among residents of GP bi-annually. This has been going on since 2009 when the first survey was conducted. The present study used the 2015 survey wave (data of adults aged 18 years and older). The questionnaire used in the survey had a total of 228 questions that covered the following aspects: type of respondent’s dwelling, how residents came to live in the study area (born in Gauteng or immigrant), details about the suburb or community where respondent resides, mode of transportation available to the respondent, access to internet services and other household information, participation in community processes, respondents’ view about their own life, employment and work opportunities, issues related to crime and safety, health-related issues, and socio-demographic aspects. Two hundred and twenty-four (224) of the 228 questions were closed, and only four (*n* = 4) were open-ended.

During data collection, the GCRO closely monitors the process on a live basis to guarantee the quality and integrity of the data that is collected. Furthermore, the GRCO enhances the quality of the data it collects by implementing rigorous checking and quality control processes [18,19]. The internal validity of the data is safeguarded by, first, conducting a pilot study. Then, the results of the pilot study are reworked into the final questionnaire (Ask Africa, 2016) [20]. For the 2015 QoL wave, the questions remained the same as the previous wave.

The face-to-face Computer Aided Personal Interviewing (CAPI) method that makes use of portable electronic devices (e.g., tablets) was employed to collect data from the field. During the structured interviews, the interviewers read survey questions to the participants and the responses are captured using the electronic devices [20].

The South African Multidimensional Poverty Index (SAMPI) included in this study was developed by Statistics South Africa. The latter used the 2011 South African census data to compute the SAMPI. This was later merged with the QoL survey data and included as a fixed effect variable.

### 3.4. Data management and Data Analysis

#### 3.4.1. Data Management

There were 23 variables included in this study, of which 22 were extracted from the QoL data, and the SAMPI was computed from the SA 2011 census data. The nature of the variables and their anticipated effects on the outcome (growing food or vegetables) are presented in Table S1.

#### 3.4.2. Analytical Approach and Data Analysis 

The data were analysed using the statistical software package Stata IC V.15.1 (StataCorp, College Station, TX, USA). The dataset was first assessed for duplicates and missing information before analysis commenced. None of the variables had missing information. Some of the variables were recorded into fewer levels to render them suitable for analysis (Table S1).

Given that the outcome (growing food or vegetables) was reclassified into a binary variable (Growing versus Not growing), a binary logistic regression was adopted to assess the correlation between a set of independent (explanatory) variables and the binary dependent variable (Not growing = 0 and Growing = 1) [21].

Unweighted descriptive statistics (the proportion of respondents growing food by socio-economic and demographic variables) were computed for the whole sample. 

Multicollinearity between independent variables makes it difficult to interpret the model and/or leads to over fitting. In view of this, we tested for correlation between the independent variables in the regression model by performing a multicollinearity test. None of the independent variables were correlated to one another. 

The mathematical equation for the binary logistic regression is as follows:

(1) $$\ell =\log_{b}\frac{p}{1-p}=\beta_{0}+\beta_{1}x_{1}+\beta_{2}x_{2}$$

where

*ℓ* is the log-odds,
*b* is the base of the logarithm, and
*βi* are parameters of the model.

The above formula shows that once β_i_ is fixed, we can compute the log odds that Y = 1 for a given observation. Therefore, the logistic regression enables us to compute the probability p that Y = 1 given a set of observations (X_1_, X_2_ … X_i_).

Model building was done in two phases. The first phase involved assessing simple associations to identify potential predictors associated with the outcome at a generous α ≤ 0.20. Variables that were significantly associated with the outcome in the univariable model were included in the multivariable model. Then, this was followed by fitting a multivariable logistic regression model using the manual backwards selection method. The level of significance for the multivariable analysis was set at α ≤ 0.05.

We assessed confounding by comparing the change in model coefficients with and without the suspected confounders. Where the removal of a suspected confounding variable resulted in a ≥20% change in the coefficient of any variable in the model or changed the significance of the fit of the model, the variable was considered a confounder and thus was retained in the model regardless of whether it was significantly associated with the outcome variable or not. 

Possible interactions were tested in the final main effects model. However, no interaction term reached statistical significance (*p* ≤ 0.05). Therefore, interaction terms were not retained in the final model. Parameter estimates (Coefficients) and their 95% confidence intervals were computed for variables included in the final model. The Hosmer–Lemeshow goodness of fit test was used to assess model fit.

## 4. Results

### 4.1. Descriptive Statistics

Summary statistics of adults (≥18 years old) in the Quality of Life survey, 2015 wave, Unweighted are presented in Table 1 and Table 2.

Overall, the results presented in Table 1 show that most people in Gauteng did not engage in growing food gardens. We observed that based on the demographic profile of the respondents, over 80% for each variable did not grow food or vegetables. For example, based on the place of birth, irrespective of where the respondent was born, over 80% indicated that they did not grow food or vegetables. This was also true for all the other variables (Table 1). 

Respondents who indicated that they had migrated to Gauteng, from other provinces within South Africa, were slightly more likely to grow food or vegetables compared to those who said they were born in Gauteng (13.71% versus 12.37%) or had migrated to Gauteng from other countries (13.7% versus 12.37%).

With respect to population groupings/race, more Blacks (13.40%) indicated that they grow food or vegetables compared to the other racial groups. Female respondents, compared to their male counterparts, were more likely to own a home food garden (13.42% versus 11.99%). Based on age, the likelihood of participating in growing food or vegetables increased with increasing age, with the age group 65 years and older more likely to grow food or vegetables (17.86%) as compared to the other age groups.

Respondents with no education (18.13%) and those who had completed primary schooling (17.08%) were the most likely groups to grow food or vegetables in their homesteads; while those who had more than matric (10.74%) and those who were captured as unspecified (8.47%) were less likely to get involved in growing food or vegetables.

The respondents who reported poor SRH were more likely to grow food or vegetables in their homesteads as compared to those who rated their health as being good (17.24% versus 12.34%). In response to the question, “Does your health prevent you from doing daily work?”, respondents who indicated that their health always prevents them from doing their work had a higher proportion or people (20.00%) who grew food or vegetables. Meanwhile, the category of those whose health never prevents them from doing their daily work had the lowest proportion (10.81%) of people growing food or vegetables.

Assessment of a food garden by socio-economic variables are presented in Table 2. As we observed for the demographic variables, we noted that for each variable, over 80% of the respondents did not grow food or vegetables. The assessment of those who grew food or vegetables showed that respondents who resided in the areas classified as 4th SAMPI Quartile were more likely to grow food or vegetables compared to those who resided in areas that fall in the 2nd and 1st Quartiles (13.83% versus 12.32% and 13.83% versus 12.20%, respectively). Respondents from areas that fall in the 3rd Quartile of the SAMPI were the least likely (11.10%) to grow food or vegetables.

Considering ownership of the dwelling place of the respondent, we observed that respondents who resided in houses built and offered to residents at no cost under the Rural Development Programme (17.91%) were more likely to grow food or vegetables as compared to those who lived in other types of accommodation. Meanwhile, respondents who lived in rented properties had the lowest number of people participating in growing food or vegetables (Table 2). 

Larger households, with seven (*n* = 7) or more people sharing the dwelling, were more likely to own a home food garden (15.03%) compared to the smaller households. Households with the least number of people sharing the dwelling (1–3 people) had the lowest proportion of people (12.15%) engaging in home gardening.

Families that have children participating in school feeding schemes were more likely to grow food or vegetables (16.37%) compared to those families that do not have children participating in a school feeding scheme (11.97%). Based on the question of whether it is important to protect the environment, participants who indicated that they neither agree nor disagree (14.67%), disagree (14.78%), and strongly disagree (13.56%) that it is important to look after the environment had more people engaged in growing food or vegetables compared to those who said that they agree (12.22%) and strongly agree (12.61%) with the statement.

Respondents who indicated that they had children who often (17.82%), always (17.27%) or sometimes (16.75%) missed meals were more likely to get involved in home gardening compared to those that said that the children in the household never (12.68%) or seldom (15.40%) missed a meal.

Similar to the case with children, households that had an adult or the respondent who had often (18.15%), always (16.36%), or sometimes (15.68%) missed meals in the past year were more likely to get involved in home gardens compared to the respondents who said they never missed a meal in the past year.

Households that had a person receiving a social grant were more likely to participate in growing food or vegetables, as compared to those that said that they did not have a member who was a recipient of a government social grant (15.28% versus 10.90%). Based on the employment status, respondents who were classified as “Other” were more likely to grow food or vegetables compared to the employed category (13.85% versus 11.97%).

Respondents whose source of water was not more than 20 m away were more likely to own a home food garden compared to respondents whose water source was located more than 20 m away (16.33% versus 15.31%). Meanwhile, the category that indicated that they had had their water cut off for non-payment in the past year had more people (16.23%) growing food or vegetables compared to the category that indicated that their water had not been cut off due to non-payment in the same period.

Respondents whose main source of water was boreholes/wells (31.16%), rainwater or tank (23.08%), and rivers or dams (20.83%) had the highest proportion of people involved in growing food or vegetables. Those who had piped water as their main source of water were the least likely to participate in growing food and vegetables. 

Respondents who indicated that they participated in activities of a club or social group in the past year had more people (14.47%) who participated in growing food or vegetables, as compared to those who had not participated in activities of a club or social group (11.41%).

#### Inferential Statistics

Results of the investigation of the factors correlated with growing food (i.e., owning a home garden) are presented in Table 3. The results show that respondents, who were residents in an area that fell under the 4th Quartile of the SAMPI (the poorest areas), were significantly (*p* = 0.002) more likely (Coeff = 0.152; 95%CI (Confidence Interval): 0.056–0.248) to grow food compared to those who lived in the area that belonged to the 1st Quartile of the SAMPI (richest areas). No association was observed between the other levels of the SAMPI (2nd to 3rd) with growing food (*p* > 0.05).

Respondents who rented from private individuals (Coeff = −0.667; 95%CI: −0.802–−0.531) or the government (Coeff = −0.254; 95%CI: −0.442–−0.066) were significantly less likely (*p* < 0.05) to grow vegetables or food compared to those who owned the dwelling. However, if respondents dwelled in a free RDP house, they were significantly (*p* < 0.05) more likely to grow food in their backyard (Coeff = 0.288; 95%CI: 0.181 0.394). There was no association between other types of dwelling ownership and growing food (*p* > 0.05).

Respondents whose health prevented them from doing daily work some of the time (Coeff = −0.617; 95%CI: −0.767–−0.446), hardly ever (Coeff = −0.649, 95%CI: −0.807–−0.490), and never (Coeff = −0.430; 95%CI: −0.574–−0.286) were significantly (*p* < 0.05) less likely to grow food compared to those whose health always prevented them from doing daily work. Respondents who neither agreed nor disagreed (Coeff = 0.156; 95%CI: 0.023–0.289) with the view that it was important to look after the environment were significantly (*p* < 0.05) more likely to grow food compared to those who said that they strongly agreed with the statement that it is important to look after the environment. However, if the respondents agreed, disagreed, or strongly disagreed with the statement “it is important to look after the environment”, they were significantly less likely (*p* > 0.05) to grow vegetables compared to those who strongly agreed with the statement.

If a household had an adult or a respondent who, in the past year, had skipped a meal either seldom (Coeff = 0.157; 95% CI: 0.007–0.306), sometimes (Coeff = 0.130; 95% CI: 0.013–0.246), or often (Coeff = 0.289; 95% CI: 0.034–0.544), such a household was significantly more likely to grow food or vegetables, compared to households where an adult or respondent did not skip a meal in the past year. However, there was no difference (*p* > 0.05) in the likelihood of growing food or vegetables between households with an adult or the respondent who had always skipped a meal in the past year and households in which a respondent or adult never skipped a meal in the past year.

Respondents whose main source of water was boreholes or wells (Coeff = 1.289; 95%CI: 0.969–1.608) were significantly (*p* < 0.05) more likely to grow vegetables compared to those whose main source of water was piped water. Meanwhile, households whose main source of water was rainwater/tank (Coeff = 0.486; 95%CI: −0.244–1.216), river/dams (Coeff = 0.799; 95%CI: −0.278–1.877), and water tank/truck (Coeff = 0.274; 95%CI: −0.063–0.611) were more likely to grow vegetables than households that had piped water as their main source of water. However, the difference did not reach significance (*p* > 0.05). Although households that had “Others” as the main source of water were less likely (Coeff = −0.188; 95%CI: −0.728–0.353) to grow food, compared to those that had piped water as the main source of water, the difference did not reach significance (*p* > 0.05).

Households that had a member who was receiving a social grant (Coeff = 0.092; 95%CI: 0.001–0.184) were significantly more likely to grow food compared to households that did not have a member receiving a social grant.

Whether a person migrated from another province (Coeff = 0.148; 95%CI: 0.061–0.236) or outside the country (Coeff = 0.314; 95%CI: 0.158 –0 471) to Gauteng, they were significantly (*p* < 0.05) more likely to grow vegetables compared to those who were born in Gauteng. If a household had four to six people (Coeff= −0.089; 95%CI: −0.019–0.168), it was less likely to grow food compared to households with one to three people. However, the difference did not reach significance (*p* > 0.05). Households that had seven or more people (Coeff = 0.002; 95%CI: −0.137–0.141) were more likely to grow food in their homesteads. Likewise, the difference did not reach significance (*p* > 0.05).

Households that had a respondent who participated in club activities or social activities (Coeff = 0.210; 95%CI: 0.132–0.287) were significantly (*p* < 0.05) more likely to grow food compared to households where the respondent did not participate in any club activities or social activities. Compared to Black households, all other races were less likely to grow vegetables. However, only Indians/Asians (Coeff = −0.504; 95%CI: −0.896–−0.112) were significantly (*p* < 0.05) less likely to grow vegetables compared to Blacks.

Respondents who were aged between 36 and 49 (Coeff = 0.075; 95%CI: −0.019–0.168) were more likely to grow vegetables, compared to those who were aged between 18 and 35, but the difference was not significance (*p* > 0.05). On the contrary, respondents who were aged 50−64 (Coeff = 0.271; 95%CI: 0.167–0.375) and 65 and above (Coeff = 0.441, 95%CI: 0.285–0.596) were significantly (*p* < 0.05) more likely to grow vegetables compared to those who belonged to the 18-35 age group.

Based on how the respondents rated their own health, we observed that respondents who rated their health as being good (Coeff = −0.216; 95%CI: −0.346–−0.085) were significantly (*p* < 0.05) less likely to grow food in their backyard compared to those who rated their health as being poor.

## 5. Discussion

To the best of our knowledge, this is the first study to investigate the level of participation in home gardening and factors that are correlated with growing food (home food gardening) in the whole GCRO. We observed that the level of involvement in home gardening was low in the study area. We further observed that the following factors were positively correlated with respondents owning home food gardens or growing food in their backyards: residing in poorest areas (areas that fall in the 4th Quartile of the SAMPI), in a free RDP house, borehole water/well was the main source of water for the household; belonging to a social club, having someone in the household receiving a social grant, being 65 years and older, and rating their health as poor. On the contrary, factors that were negatively correlated with growing food or vegetables included if the respondent rented from a private individual, their health status prevented the respondent from doing daily work, or whether the respondent agrees or disagrees that it is important to look after the environment.

It has been reported that in GP, the Department of Agriculture and Rural Development (GDARD) offers support to very few home garden projects, which, moreover, happen to be situated mainly in the underdeveloped and vulnerable communities [3]. Therefore, it is not surprising that very few respondents in this study participated in home gardening. Furthermore, findings of this study are consistent with what has been reported in previous studies done in other parts of South Africa and elsewhere [5,10]. For example, Philander et al. (2016) in a study conducted in Langa, Cape Town, revealed that despite the benefits of home food gardens, participation was very low at 38%. A study conducted in Myanmar also showed that only 959 households out of 3239 (29.61%) participants owned home food gardens [5], and only 16 participants of 115 (13.91%) respondents were regular participants in food gardening in a study that was conducted in the United States [22]. The low percentage of participation in home gardening has been attributed to limited agricultural inputs, land shortages, poor soils, water scarcity, and lack of knowledge and advisory services [4]. 

Statistics South Africa found that provinces that are mainly urban tend to have the lowest proportion of households participating in agricultural activities when compared to provinces that are mainly rural [12]. Furthermore, it has been shown that home food gardening is more prevalent in rural areas compared to urban areas [9,12]. This could explain why respondents who had migrated from other provinces were more likely to participate in growing food in this study. This is because as people migrate from rural areas, they usually bring along agricultural practices to the urban areas [10], as they are used to gardening back in the rural areas where they come from.

According to a study conducted by Statistics South Africa [12], households headed by Black South Africans are more likely to suffer food insecurity as compared to households headed by other races. This could explain why in this study, more Black South Africans tended to participate in home gardening compared to the other races. It is postulated that their involvement in home gardening is an attempt to improve on their food security status, and also to generate income [9].

Consistent with findings by Bongiwa and Obi (2015) and Phulkerd et al. (2020), more female respondents in this study participated in home food gardens than males. Likewise, in a study that was conducted in Benin, more women were found to own food gardens, even at a younger age, compared to their male counterparts [23]. Results observed in this study could be due to the fact that females, in addition to being more extremely vulnerable to food insecurity than their male counterparts, are also mainly responsible for household food security through food production, food processing, preservation, and preparation [24,25].

It is well known that a strong correlation exists between low educational status and food insecurity. It is postulated that education provides a buffer against food insecurity because it increases the prospects of one finding employment [10]. Some authors have even suggested that it is because people with a higher education status are likely to be employed, and as a result, they lack time to participate in home gardening [23]. This could explain why there was a high participation in growing food or vegetables (home food gardening) amongst people with low educational levels in this study. Findings of the present study are consistent with findings of a study that was conducted in the Eastern Cape province of South Africa, in which it was revealed that there was a strong relationship between home gardening and low education status of respondents [9].

More respondents who dwelled in houses built as part of the RDP (i.e., RDP houses), households with children benefiting from school feeding schemes, larger households, and those that had reported signs of food insecurity, such as skipping meals, participated in home gardens, which was expected and is an encouraging finding. This is because past studies show that there is a high prevalence of food insecurity amongst poor urban residents [12]. Furthermore, large household sizes increase the labour capacity of a household, which facilitates participation in home gardening [9].

In this study, we observed that proximity of water source (less than 20 m away) was strongly correlated with growing food or vegetables. Water is a very important resource in gardening, such that limited access to water has been identified as one of the major constraints to home food gardening [4]. When the source of water is far, collection requires manpower and, hence, doubles the workload, which negatively impacts on agricultural production and food security [26].

The fact that the probability of one growing food in the backyard of the homestead is higher if one resided in the poorest areas (areas that fall under the 4th Quartile), resided in a free RDP house, and had a member in the household received a social grant suggests that people with a poor socio-economic status are more likely to get involved in growing their own food as compared to the more affluent residents of the GP. These findings are consistent with findings of studies done in Thailand [14], Benin [23], and the Eastern Cape (South Africa) [9]. The implications of these findings are that people with low socio-economic status, in the study area, are making use of home food gardens to supplement their household food baskets and/or generate some income. This finding could also be due to the fact that GDARD offers support to very few home food garden projects and, moreover, the support that they offer is limited to underdeveloped and vulnerable communities [3].

There was a strong positive correlation between gardening and the health status of respondents. Respondents who reported that their health prevented them from doing daily work and those that rated their health as being poor were more likely to participate in gardening than other groups that reported having better health status. As has been reported in previous studies, this could be based on the belief that home gardening improves one’s health status [4,10,27]. For example, Galena et al. [4] suggest that home gardening has medicinal value and could be used to treat common ailments, such as vitamin A and iron deficiencies. Furthermore, gardening has also been found to reduce the symptoms of serious illnesses, such as cancer, strokes, dementia, allergies, and asthma by reducing pain and offering therapeutic benefits [27]. Therefore, it is possible that respondents in this study use home gardening to augment their health status.

The findings of this study also show a significant positive correlation between home gardening and age, with respondents who were 65 years and older more likely to own home food gardens as compared to younger respondents (18–35 years). Several other authors have made similar observations [9,14,23]. Apart from the fact that more older people tend to be the main primary food providers, the literature also suggests that older people are likely to have more time and skills to spend on home gardening activities than the younger age groups [14]. 

Home food gardens are mainly rain fed; however, depending on the plant, geographic area, season, and rainfall, irrigation could become necessary. This is particularly true considering climate variability, which makes rainfall unreliable [26]. Yet, as has been observed, water in urban areas tends to be scarce and can be expensive, especially for poor resource households. Therefore, it was not surprising that households that rely on boreholes for their water were positively correlated with growing their own food or vegetables compared with those that depended on piped water as the main source of water. The literature indicates that in areas where households have to purchase water, the cost can be high [26]. This combined with scarce financial resources, results in discouragement from participating in home food gardens.

Results reported here also showed a positive correlation between participating in a social club and home gardening. This finding confirms previous observations that suggested a strong association between gardening and social capital [4,7]. Through social capital, households are able to exchange knowledge, skills and gifts, thus, building integrated societies [4].

## 6. Limitation of the Study

The present study used secondary data and, so, the researchers did not have control over the kind of variables that were collected. Due to this, the analysis was limited to the variables in the data. For example, the data lacked a variable on the size of land available to the different respondents. As a result, it was not possible to investigate if land availability was a limiting factor for participating in home food gardens. The data did not include variables on the kind of crops that are grown by the respondents. Therefore, it is not possible to determine the contribution of the home food gardens to dietary variability and quality. In addition, since the data only covered Gauteng, it is not possible to generalise the findings reported here to other provinces. Lastly, this being a purely quantitative and observational study, it was not possible to establish causality of certain occurrences, such as why larger families (with seven or more people) were not significantly associated with growing food and why gender was not significantly associated with growing food. Nonetheless, the findings of this study provide baseline information upon which future studies can be based. Secondly, the findings of this study can be used by policy makers to develop policies that can help promote participation in home food gardening.

## 7. Conclusions

To the best of our knowledge, participation in home gardens and correlated factors have not been studied at provincial level in GP. This study contributes to the body of literature on the extent of home gardening and sheds light on the socio-demographic factors that are correlated with home gardening. Participation in home gardening is generally low, which suggests the failure of the current policies to promote home gardening in the province. The study identified the socio-economic and health factors that play a crucial role in participation in home gardening. Therefore, the government and other stakeholders should focus on these factors to promote home gardening and improving food security, especially amongst those of a low socio-economic status. The findings of the present study could assist policy makers to design tailor-made interventions that will promote home food gardening. Lastly, focused studies are needed to establish the cause of the generally low level of participation in home gardening, despite the reported food insecurity at the household level. Furthermore, studies are needed to investigate the type of food or vegetables grown in these home gardens as a follow up to this study. This will help clarify the role that home gardens play in the food security status of the residents of the study area.

## Figures and Tables

**Table 1:** Respondents in the Quality of Life survey data who grow food or vegetables based on their demographic profiles and health status-related variables.

Variable	Level	Growing		Not Growing		*p*-Value
		*n*	%	*n*	%	
Place of birth	Born in Gauteng	2373	12.37	16,808	87.63	0.007
	Migrated to Gauteng from another province	1167	13.71	7342	86.29	
	Migrated to Gauteng from another country	286	12.37	2026	87.63	
Population group	Blacks	3238	13.40	20,927	86.60	0.000
	Coloureds	112	9.79	1032	90.21	
	Indian/Asian	45	7.11	588	92.89	
	White	423	10.74	3515	89.26	
	Other	8	6.56	114	93.44	
Sex	Male	1673	11.99	12,280	88.01	0.000
	Female	2153	13.42	13,896	86.58	
Age of respondent	18–35 years	1580	11.24	12,477	88.76	
	36–49 years	1034	12.27	7390	87.73	
	50–64 years	844	15.46	4616	84.54	
	65 years and above	368	17.86	1693	82.14	
Education level of respondent	No education	89	18.13	402	81.87	0.000
	Primary only	563	17.08	2734	82.92	
	Secondary incomplete	1249	13.92	7724	86.08	
	Completed matric	1150	11.62	8750	88.38	
	More than matric	725	10.74	6026	89.26	
	Unspecified	50	8.47	540	91.53	
Self-rated health (SRH) of respondent	Poor health	433	17.24	2079	82.76	
	Good health	3393	12.34	24,097	87.66	
Does your health prevent you from doing daily work?	Always	345	20.00	1380	80.00	0.000
	Sometimes	864	12.71	5934	87.29	
	Hardly ever	689	10.81	5685	89.19	
	Never	1928	12.76	13177	87.24	

*n*: Number of respondents; %: proportion of respondents.

**Table 2:** Respondents in the Quality of Life survey data that grow food or vegetables based on their socio-economic profiles.

Variable	Level	Growing		Not Growing		*p*-Value
		*n*	%	*n*	%	
SAMPI	1st Quartile	1369	12.20	9856	87.80	0.005
	2nd Quartile	923	12.32	6566	87.68	
	3rd Quartile	495	11.10	3283	86.90	
	4th Quartile	1039	13.83	6471	86.17	
Dwelling ownership	Own dwelling	1639	12.96	11,005	87.04	0.000
	Renting/private	408	6.70	5613	93.22	
	Renting/government	158	10.84	1300	89.16	
	Free RDP house ^1^	788	17.91	3613	82.09	
	Transferred deed	186	14.17	1127	85.83	
	Rent free	368	15.17	2058	84.83	
	Occupy vacant dwelling	149	15.50	812	84.50	
	Other	130	16.71	648	83.29	
Type of dwelling	Formal	3236	12.62	22,407	87.38	0.000
	Informal	559	14.33	3343	85.67	
	Other	31	6.78	426	93.22	
Number of people living in household	1–3 people	1994	12.15	14,424	87.85	0.000
	4–6 people	1405	13.08	9339	86.92	
	7 and more people	427	15.03	2414	84.97	
Children belong to school feeding scheme	No	2011	11.97	14,791	88.03	0.000
	Yes	1225	16.37	6257	83.63	
	N/A	590	10.32	5128	89.68	
Important to look after environment?	Strongly agree	1446	12.61	10,022	87.39	0.001
	Agree	1683	12.22	12,088	87.78	
	Neither agree nor disagree	459	14.67	2669	85.33	
	Disagree	198	14.78	1142	85.22	
	Strongly disagree	40	13.56	255	86.44	
Children skipped meal in past year	Never	2562	12.68	17,645	87.32	0.000
	Seldom	210	15.77	1122	84.23	
	Sometimes	381	16.75	1894	83.25	
	Often	59	17.82	272	82.18	
	Always	24	17.27	115	82.73	
	No children in household	598	10.32	5128	89.68	
Adult or respondent missed a meal in past year	Never	2904	12.01	21,275	87.99	0.000
	Seldom	279	15.40	1533	84.60	
	Sometimes	516	15.68	2774	84.32	
	Often	92	18.15	415	81.85	
	Always	35	16.36	179	83.64	
Water source > 20 metres	No	249	16.33	1276	83.67	0.000
	Yes	154	15.31	852	84.69	
	N/A	3423	12.46	24,048	87.54	
Water cut off for non-payment	No	3512	12.51	24,555	87.49	0.000
	Yes	314	16.23	1621	83.77	
Main source of water	Piped water	3648	12.50	25,532	87.50	0.000
	Borehole/well	91	31.16	201	68.84	
	Rainwater/tank	12	23.08	40	76.92	
	River/dams	5	20.83	19	79.17	
	Water tank/truck	54	17.70	251	82.30	
	Other	16	10.74	133	89.26	
Someone in household receives a social grant	No	1885	10.90	15,411	89.10	0.000
	Yes	1941	15.28	10,765	84.72	
Employment status	Employed	1744	11.97	12,827	88.03	0.000
	Unemployed	1073	13.17	7075	86.83	
	Other	1009	13.85	6274	86.15	
In the past year, participated in activities of club or social group	No, none at all	1926	11.41	14,948	88.59	0.000
	Yes, any club or social group	1900	14.47	11,228	85.53	

^1^ Government subsidy housing that is commonly known as RDP houses. The houses are built by the government and are given to low income families. These houses are owned, and not rented by the beneficiaries.

**Table 3:** Factors correlated with growing of food among residents of Gauteng City Region Observatory in South Africa (2015 Quality of Life survey).

Variable	Parameter Estimates					* H-L Gof *p*-Value
Growing Food	Coeff	SE	# *p*-Value	95%CI		0.072
SAMPI ^x^						
1st Quartile	ref					
2nd Quartile	0.001	0.050	0.977	−0.098	0.100	
3rd Quartile	0.086	0.063	0.179	−0.040	0.211	
4th Quartile	0.152	0.049	0.002	0.056	0.248	
Do you own a place of dwelling?						
Own dwelling	Ref					
Renting from private	−0.667	0.069	0.000	−0.802	−0.531	
Renting from government	−0.254	0.095	0.008	−0.442	−0.066	
Free RDP house	0.288	0.054	0.000	0.181	0.394	
Transfer deed	−0.085	0.092	0.359	−0.266	0.096	
Rent free	0.060	0.075	0.426	−0.088	0.207	
Occupy vacant dwelling	0.096	0.104	0.359	−0.109	0.300	
Other	0.174	0.111	0.119	−0.044	0.392	
My health prevents daily work						
Always	Ref					
Some of the time	−0.617	0.076	0.000	−0.767	−0.446	
Hardly ever	−0.649	0.081	0.000	−0.807	−0.490	
Never	−0.430	0.074	0.000	−0.574	−0.286	
Important to look after environment						
Strongly agree	Ref					
Agree	−0.053	0.043	0.216	−0.138	0.031	
Neither agree nor disagree	0.156	0.068	0.022	0.023	0.289	
Disagree	0.131	0.088	0.139	−0.043	0.304	
Strongly disagree	0.049	0.183	0.788	−0.310	0.408	
Past year, adult skipped a meal						
Never	Ref					
Seldom	0.157	0.076	0.040	0.007	0.306	
Sometimes	0.130	0.059	0.029	0.014	0.246	
Often	0.289	0.130	0.027	0.034	0.544	
Always	0.130	0.200	0.516	−0.262	0.523	
Main source of water						
Piped water	Ref					
Borehole/well	1.289	0.163	0.000	0.969	1.608	
Rainwater/tank	0.486	0.372	0.192	−0.244	1.216	
River/dams	0.799	0.550	0.146	−0.278	1.877	
Water tank/truck	0.274	0.172	0.111	−0.063	0.611	
Other	−0.188	0.276	0.497	−0.728	0.353	
A person in household receives a social grant						
No	Ref					
Yes	0.092	0.047	0.048	0.001	0.184	
Birthplace						
Gauteng	Ref					
Migrant from another province	0.148	0.045	0.001	0.061	0.236	
Migrant from another country	0.314	0.800	0.000	0.158	0.471	
Number of people in household						
1–3 people	Ref					
4–6 people	−0.089	0.046	0.056	−0.179	0.002	
7 and above	0.002	0.071	0.980	−0.137	0.141	
Membership of club or social club						
No, not any club or social	Ref					
Yes, any club or social club	0.210	0.039	0.000	0.132	0.287	
Population grouping/Race						
Blacks	Ref					
Coloured	−0.226	0.121	0.062	−0.463	−0.012	
Indian/Asian	−0.504	0.200	0.012	−0.896	−0.112	
White	−0.061	0.069	0.373	−0.196	0.073	
Other	−0.386	0.700	0.580	−1.754	0.981	
Age group						
18–35	Ref					
36–49	0.075	0.048	0.117	−0.019	0.168	
50–64	0.271	0.053	0.000	0.167	0.375	
65 and above	0.441	0.079	0.000	0.285	0.596	
Self-rated health						
Poor SRH	Ref					
Good SRH	−0.216	0.067	0.001	−0.346	−0.085	

x: South African Multidimensional Poverty Index; *: Hosmer–Lemeshow goodness of fit test *p*-value; CI: 95% Confidence Interval; #: Fisher’s exact *p*-values; Coeff: Coefficients (Parameter estimates).

## Data Availability

Restrictions apply to the availability of these data. Data was obtained from a third party [Gauteng City-Region Observatory] but are available from the owner on reasonable request to [gcro@gcro.ac.za] with permission of [GCRO].

## Supplementary Materials

The following are available online at https://www.mdpi.com/1660-4601/18/5/2737/s1, Table S1. Dependent and independent variables extracted from the 2015 QoL data that was included in the binary logistic regression model.

## Abbreviations

GDARD	Gauteng Department of Agriculture and Rural Development
GP	Gauteng Province
RDP	Rural Development Programme
GCRO	Gauteng City Region Observatory
QoL	Quality of Life survey
SAMPI	South African Multidimensional Poverty Index
