# Supplementary material for: Factors Correlated with Home Gardening in Gauteng Province, South Africa
Source: Int J Environ Res Public Health. 2021 Mar 8;18(5):2737. doi: 10.3390/ijerph18052737 (PMC7967462; doi:10.3390/ijerph18052737)
Supplement: Supplementary file 1 [file ijerph-18-02737-s001.pdf]

Table S1. Dependent and independent variables extracted from the 2015 QoL data that was included in the binary logistic regression model.

Variable extracted	Description	Expected effect
Y_i =Grow food or vegetables	Outcome variable<br>Categorical (Recorded: No =0; Yes=1)	
X_1=SRH-Self Rated Health: Self rated Health	Categorical (recorded to two levels: poor health=0, good health=1)	Positive
X_2=Age	Continuous (Recorded into categories: 18-35=1; 36-49=2; 50-64=3; ≥65=4)	Positive
X_3=Race	Categorical (Recorded: blacks=1; coloureds=2; Asians/Indians=3; whites=4; others=5	Positive
X_4=Gender	Binary (male=0; female=1)	Positive
X_5=Birthplace (Born in Gauteng or immigrated to Gauteng)	Categorical (Born in Gauteng=1; immigrated from province in South Africa=2, immigrated from outside SA=3)	Positive
X_6=No in household (size of household)	Numerical (Recorded: 1-3=1; 4-6=2; 7 and more=3	Positive
X_7=Employment status	Categorical (Recorded unemployed=0; employed=1; other=3)	Positive
X_8=Education Level	Categorical (recorded: no education=1; primary education only=2; secondary incomplete=3; matric=4; more than matric=5; other=6	Positive
X_9=Grant Recipient	Binary (no=0; yes=1)	Positive
X_{10}=Membership of a club	Binary (no=0; yes=1)	Positive
X_{11}=Main source of water	Categorical (Recorded: (Piped water=1; borehole/well=2; rainwater/tanks=3; River/dams=4; Water tank/truck=5; other=6)	Positive
X_{12}=Water cut off in previous year?	Binary (no=0; Yes=1)	Positive
X_{13}=Water source more than 20 M away?	Binary (No =0; Yes=1)	positive
X_{14}=Registration of house with office	Binary (No=0; Yes=1)	Positive
X_{15}=Dwelling	Categorical (recorded: own dwelling=1, rent/private=2; rent/government=3; Free RDP=4; Deed transferred=5; rent free=6; occupy vacant land=7; other=8)	Positive
X_{16}=Type of dwelling	Categorical(recorded: formal=1; informal=2; other=3)	Positive
X_{17}=Do children belong to school feeing scheme?	Binary (no=0; yes=1)	Positive
X_{18}=Did ownership adult in household skip mean in past year?	Categorical (Recorded: never=1, seldom=2 sometimes=3; often=4; always=5	Positive
X_{19}=Did child in home skip meal in past year?	Categorical (Recorded: never=1, seldom=2 sometimes=3; often=4; always=5	Positive
X_{20}=Does your health prevent you from doing work?	Likert scale (always=1; some of the times=2; hardly ever=3; never= 4)	Negative
X_{21}=Is it important to protect the environment?	Likert scale (strongly agrees=1; agrees=2; neither agrees nor disagrees=3; disagrees=4' strongly disagrees=5	Positive
X_{22}=SAMPI	Continuous (Create 4 quartiles)	Positive
